# Does Cardiorespiratory Fitness Influence the Effect of Acute Aerobic Exercise on Executive Function?

**DOI:** 10.3389/fnhum.2020.569010

**Published:** 2020-10-06

**Authors:** Jie Cui, Liye Zou, Fabian Herold, Qian Yu, Can Jiao, Yanjie Zhang, Xinli Chi, Notger G. Müller, Stephane Perrey, Lin Li, Chenbo Wang

**Affiliations:** ^1^College of Physical Education and Health, East China Normal University, Shanghai, China; ^2^Key Laboratory of Adolescent Health Assessment and Exercise Intervention of Ministry of Education, East China Normal University, Shanghai, China; ^3^Exercise and Mental Health Laboratory, School of Psychology, Shenzhen University, Shenzhen, China; ^4^Research Group Neuroprotection, German Center for Neurodegenerative Diseases, Magdeburg, Germany; ^5^EuroMov Digital Health in Motion, Univ Montpellier, IMT Mines Ales, Montpellier, France; ^6^Key Laboratory of Brain Functional Genomics, MOE & STCSM, School of Psychology and Cognitive Science, East China Normal University, Shanghai, China; ^7^Institute of Brain and Education Innovation, East China Normal University, Shanghai, China

**Keywords:** executive function, acute aerobic exercise, cardiorespiratory fitness, Stroop task, fMRI

## Abstract

**Background:**

The beneficial effects of acute exercise on executive function have been well-documented, but the influence of cardiorespiratory fitness on this effect requires further investigations, especially using imaging technique. This study aimed to examine the effects of cardiorespiratory fitness on acute exercise-induced changes on behavioral performance and on functional brain activation.

**Method:**

Based on their cardiorespiratory fitness level, 62 participants ranked in the top and bottom of the maximum oxygen consumption (VO_2_ max) were finally selected and allocated to high-fit group or low-fit group. Both groups were asked to complete the Stroop task after 30 min of aerobic exercise and chair-seated rest (control session). Among them, 26 participants were randomly selected and asked to undergo the Functional Magnetic Resonance Imaging (fMRI).

**Results:**

Behavioral results showed that individuals responded significantly faster after exercise than those in the control session. The fMRI results revealed a significant interaction effects of Group by Session in brain regions including anterior cingulate cortex (ACC) and bilateral dorsal lateral prefrontal cortex (DLPFC). For the ACC, activation in the high-fit group was significantly decreased after aerobic exercise compared to those in the control session; whereas an increased activation was noticed in the low-fit group. Regarding to the bilateral DLPFC, activation in high-fit group was significantly decreased after exercise compared to those in the control session, while no significant differences were found in the low-fit group. In addition, for the post-exercise session, a significant positive correlations between activation of the ACC and left DLPFC in the high-fit group was observed. There was a significant negative correlation between activation of the ACC and reaction time in the congruent condition after exercise in the low-fit group.

**Conclusion:**

Findings further clarify the neurophysiological processes of acute exercise-induced changes in cognitive performance as they suggest that cardiorespiratory fitness is an important factor which influences changes in brain activation patterns in response to acute aerobic exercises.

## Introduction

The beneficial effects of acute exercise have been well documented in the literature for various cognitive domains such as memory (Etnier et al., 2016; Dilley et al., 2019; Loprinzi et al., 2019; Waters et al., 2020; Zou et al., 2020), attention (Chang et al., 2017; de Sousa et al., 2019), and executive function (EF) (Verburgh et al., 2014; Chang et al., 2017) and even under hypoxia (Jung et al., 2020). Furthermore, it is generally agreed that EF is more likely to be positively influenced by acute exercise than other cognitive domains (McMorris and Hale, 2012). Indeed, there is some evidence showing that the largest acute exercise-induced benefits occur for EFs (Chang et al., 2014b). However, the effect of acute exercise on the EF was differently observed among different individuals.

Some physiological factors might attribute to the exercise-induced benefits on EF. It has also been reported that cardiorespiratory fitness (CRF) level is related to the performance in EFs (Colcombe and Kramer, 2003; Chang et al., 2012; Kramer and Colcombe, 2018) and several studies observed that higher levels of CRF are linked to better performance in EFs (Dupuy et al., 2015; Scott et al., 2016; Mekari et al., 2019). In line with this, some research findings suggest that individuals with a higher level of CRF benefited more from acute aerobic exercise than those with a relative low CRF level (Chang et al., 2015a; Hogan et al., 2015; Tsai et al., 2016). In addition, Chang et al. (2014a) observed a curvilinear relationship between CRF level and task performance implying that a moderate CRF level is associated with superior EF. However, results of a recently published meta-analysis indicated that both individuals with relative low CRF level and individuals with a relative high CRF benefit from an acute bout of moderate-intensity aerobic exercises (Ludyga et al., 2016). In summary, the available evidence suggests that it is still not fully clear whether the effects of acute exercises on EF are influenced by the individual CRF level. This, in turn, necessitates the need for further research in this direction.

In this context, neuroimaging techniques such as fMRI may help us to further elucidate the inconsistent findings regarding the effects of CRF level on acute exercise-induced changes on cognitive performance. While a number of studies have found that acute aerobic exercise at a moderate intensity has the largest effects on the EF (Verburgh et al., 2014; Chang et al., 2015b; Mehren et al., 2019), only a few neuroimaging studies using fMRI have investigated the role of CRF level on acute exercise-induced changes in cognitive performance and functional brain activation patterns (Herold et al., 2020). For instance, Li et al. (2019) examined the effect of CRF level on acute exercise-induced changes in working memory performance by using the N-back task and assessed brain activation patterns with fMRI. In this study, significant differences in the activation of the cerebellum and anterior cingulate cortex (ACC) were observed between individuals with relatively low and high CRF level (Li et al., 2019). Furthermore, Mehren et al. (2019) noticed correlations between CRF level and acute exercise-induced changes in functional brain activation patterns in the Go-No-Go task. In summary, the above-mentioned findings of fMRI studies support the view that the effects of acute aerobic exercise may be fitness-dependent and functional brain activations are sensitive to the differences in the individual fitness level. However, a recent systematic review which summarized the findings of acute exercise studies concluded that further research investigating the influence of different CRF levels on functional brain activation patterns is necessary to broaden our understanding of exercise-cognition interaction (Herold et al., 2020).

Hence, the current study aimed to investigate the effect of different CRF levels on acute exercise-induced changes in EFs and corresponding changes in functional brain activation patterns. Different from the previous work focusing on the working memory aspect of the EF by adopting the N-back paradigm (Li et al., 2019), the current study probed the inhibit control aspect of the EF with the well-established Stroop task (Stroop, 1935; Etnier and Chang, 2009) and assessed functional brain activation patterns by fMRI. Regarding to brain areas responsible for inhibit control, the dorsolateral prefrontal cortex (DLPFC) and the ACC are recognized as two key regions (Fuster, 2000; Turner and Spreng, 2012). Thus, we hypothesized that acute aerobic exercise promotes the inhibit control ability of the EF by changing the behavioral performance and its relevant neural activities in the ACC and DLPFC; and this modulation is different between high and low cardiorespiratory fitness groups.

## Materials and Methods

### Participants

In the current study, a total of 115 healthy female college students were initially recruited. Pre-determined inclusion criteria were as follows: (1) right-handedness, (2) no use of psychotropic drugs, (3) no MRI contraindications, (4) neither menstruating nor pregnant. To ensure that all participants were able to independently perform the CRF test, the Health Screening Questionnaire (HSQ) and the Physical Activity Readiness Questionnaire (PAR-Q) were collected prior to their participation. Individuals with a history of physical illness, neurological disease, substance abuse, and/or any other illnesses were excluded from this study. The gender of the participants was restricted to avoid potential confounders of neural activation (Dimech et al., 2019). In addition, all participants were also asked whether they had gotten sufficient sleep and not drunk alcoholic or caffeinated beverages within 24 h before the beginning of the CRF test. The study protocol was approved by the Institutional Review Board of the East China Normal University and all study procedures met the guideline of the Declaration of Helsinki. All participants had signed their informed consent and received compensation of 100 RMB after completing the entire experiment.

This study aimed to examine the effects of fitness level on acute exercise-induced changes in EF. For this purpose, among all the eligible participants (*n* = 115), 31 participants were arranged into a higher fitness group and another set of 31 were arranged into a lower fitness group based on the CRF assessment. Among these 62 participants, 26 of them were randomly selected and asked to undergo the fMRI. Data of two participants were removed due to higher head motion artifacts, which ultimately led to 12 HF participants and 12 LF participants.

### Experimental Procedure

**FIGURE 1:**
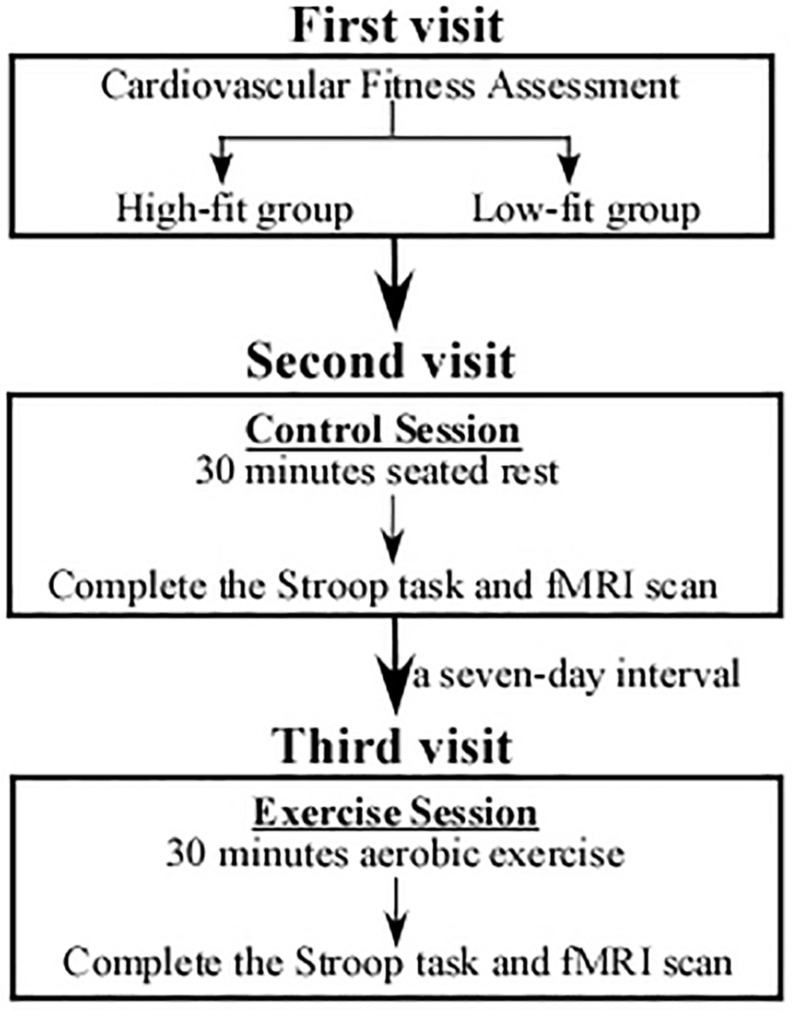
An illustration of the study procedure. During the first visit, participants were performed with a cardiorespiratory fitness assessment and divided into a High-fit group (*n* = 31) and a Low-fit group (*n* = 31). Both the HF and LF groups underwent chair-seated rest (control session) in the second visit and carried out acute aerobic exercise (experimental session) in the third visit. The order of the two visits was counterbalanced among the participants.

The experimental procedure is displayed in Figure 1. During the first visit, a total of 115 volunteers were asked to complete demographic data and questionnaires, followed by their CRF assessment with the maximum oxygen consumption (VO_2_max) that was used to determine two groups. Volunteers placed in the top and bottom 27% of the VO_2_max were finally arranged into a higher fitness group (HF, *n* = 31) and a lower fitness group (LF, *n* = 31), respectively.

During the second visit, both HF and LF groups underwent 30-min chair-seated rest (control session), followed by the third visit in which the two groups were asked to carry out 30-min acute aerobic exercise (experimental session). The two visits were counterbalanced; that is, half of the participants underwent the exercise session in the second visit and carry out the control session in the third visit. The two visits/sessions were separated by a 7-day interval. Notably, during the exercise session, only participants who completed exercise protocol were asked to rest for 15 min in a quiet room till their heart rate (HR) returned to less than 85 beats per minute (bpm). And then they were asked to perform the Stroop task and fMRI scan. All participants went through several practice trials of the Stroop task, and then the formal procedure took 10 min and 40 s.

### Cardiorespiratory Fitness Assessment

Cardiorespiratory fitness levels were indicated by their VO_2_max (ml/kg/min), measured on a Monark cycle ergometer during a YMCA protocol (Monark 839E, Sweden) (Golding, 2000). Previous work has indicated that the YMCA cycle test provides a valid and reliable estimate of VO_2_max in healthy individuals (Beekley et al., 2004; McArdle et al., 2010). This test was commonly used in studies similar to age group in the present study (Siconolfi et al., 1982; Marks et al., 1999; Zhang and Ye, 2005; Bell et al., 2017; Shi et al., 2018). In this protocol, the participants performed consecutive stages, each with increasing force, and lasting for 3 min. The participants started the test at a force of 10 N and a pedaling rate of about 50 rpm. The heart rate was continuously monitored throughout the GXT using a Polar heart rate monitor (Suunto t6, Serial No. 60502677, Finland). Participants were instructed to sit quietly for 10 min until their HR settled at their resting HR, and then they began the test. Participants who ranked in the top and bottom 27% of VO_2_ max were stratified into the HF (54.28 ± 6.32 ml/kg/min) and LF (38.73 ± 4.06 ml/kg/min) groups, respectively.

### Exercise Intervention Program

Entire exercise protocol lasted 30 min in total; that is, a 5-min warm-up was carried out, followed by 25-min cycling at a moderate intensity. During the warm-up period, participants were required to maintain their HR at approximately 120 bpm. Then participants underwent 25-min exercise on the cycle ergometer with target HR. More specifically, the target HR was calculated by the Karvonen formula: Target HR = ([HRmax – HR rest] × % intensity) + HR rest and HRmax = 220 – age. Further, the exercise intensity throughout its entire training session was fixed at 60–69%, which reflects moderate-intensity exercise. For security reasons, the entire exercise session was administered and monitored by a doctoral student in exercise science major. In the control session, the participants attended a 30-min chair-seated rest.

### Stroop Task

Executive function (EF) was measured using the Stroop Task in an event-related design. For the Stroop task, participants were required to distinguish a color-word stimulus under a conflict condition. Specifically, the Stroop test consists of congruent trials, in which four-color words (red, yellow, blue, and green in Chinese) are presented in the ink of the color indicated by the word, and incongruent trials, in which the four color words are presented in ink of a non-matching color. This task had two blocks and each block involved 32 trials, including 16 congruent and 16 incongruent trials. All trials in each block were presented in random order. In each trial, the stimulus (letter) was randomly presented in the center of the screen for 2000 ms, which was preceded by a fixation cross. Each participant was required to identify the color of the letter but have to ignore the semantic meaning by pressing a correct button in the keyboard. On the keyboards, the keys that correspond to the color of red, yellow, blue and green are C, V, N, and M respectively. The participants were instructed to respond as quickly and accurately as possible within 2000 ms. After that, inter-Stimulus Interval was randomized with a period of 2000, 4000, 6000, 8000, or 10000 ms to avoid anticipatory strategies. Before the formal experiment, the participants were asked to practice the task to familiarize with the task performance. The total experiment duration was 640 s.

### fMRI Data Acquisition and Image Processing

MRI scanning was carried out on a 3T Siemens scanner at the Functional MRI Lab (the East China Normal University, Shanghai, China). Higher resolution structural images were acquired using a magnetization-prepared, rapid-gradient echo, three-dimensional, T1- weighted sequence [TR = 2530 ms, TE = 2.34 ms, T1 = 1100 ms, flip angle (FA) = 7°, thickness = 1 mm, field of view (FOV) = 256 × 256 mm^2^, voxel size = 1.0 mm × 1.0 mm × 1.0 mm). The functional images were acquired using a gradient-echo, echo-planar imaging (EPI) sequence (TR = 2000 ms, TE = 30 ms, FA = 90, slice thickness = 3.75 mm, FOV = 192 mm × 192 mm and voxel size = 3.0 mm × 3.0 mm × 3.75 mm. Functional image preprocessing was carried out using SPM8. The first five images of each scan were excluded to account for T1-stabilization effects. For each participant, the EPI images were slice-time corrected and realigned to the first image, followed by normalization to the standard Montreal Neurological Institute EPI template. A higher-pass filter with a cutoff period of 128 s was selected. Additionally, spatial smoothing was achieved using a Gaussian kernel (8 mm^3^ full width at half maximum).

### Statistical Analysis

Descriptive data and behavioral outcomes were analyzed using the SPSS 23 statistical packages (IBM Corporation, Armonk, NY, United States). Specifically, all descriptive data (age, height, weight, body mass index, HR, and CRF) were analyzed to summarize the characteristics of the participants. The independent *t*-test was further employed to determine whether significant differences existed in those characteristics between HF and LF groups. Behavioral performances in reaction time (RT) and accuracy for the Stroop task were analyzed with a repeated-measures ANOVA in a general linear model to examine the interaction effects of Session (post-exercise and post-rest) and Group (HF and LF) in the executive performance. *Post hoc* analysis with Bonferroni correction or LSD was performed to determine if a significant difference existed in EF between HF and LF groups.

The fMRI scans were acquired during the Stroop task in an event-related design. The fMRI data were analyzed using the software toolbox SPM8^1^, which included the whole brain-analysis and ROI analysis. At the first level, two conditions were defined (congruent/incongruent). They were modeled using a canonical hemodynamic response function. We chosen the onset of the stimulus as the onset tome point and the RT from the stimulus onset to button press as the duration. Six regressors modeling movement-related variance, one modeling the fixation during the task and one modeling the overall mean were also employed in the design matrix. A general linear model analysis created two contrast images for each participant summarizing differences of interest (congruent > rest, incongruent > rest). The two first-level contrast images from each participant were then analyzed at the second level employing a random-effects model. We used *F*-contrast to analyze the Session × Group interaction effect. All areas of activation in whole brain analyses were defined using a threshold of *p* < 0.05, which was corrected by a combined voxel-intensity and cluster-size threshold of single voxel *p* < 0.001 and an extent threshold *k* > 22 voxels based on the Monte-Carlo simulation (1000 iterations) (Ledberg et al., 1998; Slotnick et al., 2003). However, we admitted that this threshold (requiring *F* > 11.5) was relatively looser than the FWE corrected *p* < 0.05 (requiring *F* > 23.5) implemented in SPM. ROI analysis was conducted to examine the effect of acute aerobic exercise on EF between HF group and LF group with post hoc comparisons. As the prefrontal cortex has been widely recognized as key structure for EF performance (Miller and Cohen, 2001; Funahashi and Andreau, 2013), three ROIs in the PFC were selected based on a previous study (Fuster, 2000; Botvinick et al., 2004; Turner and Spreng, 2012). These ROIs were defined as spheres with a radius of 6 mm centered at −4/8/26 (ACC), −36/26/50 (left DLPFC), and 56/22/12 (right DLPFC) using MarsBaR^2^. Beta values of incongruent and congruent conditions were extracted in contrast with fixation. These values were then subjected to a repeated-measures analysis of variance (ANOVA) with Session (post-exercise and post-rest) and Group (HF and LF) using the SPSS 23 (IBM Corporation, Armonk, NY, United States). Pearson correlation analysis was conducted to explore the relationship between ACC and DLPFC in both HF group and LF group. Partial correlation was performed to investigate associations between brain activation and behavioral performance in terms of post-exercise session when controlling for their baseline data. The significance level was set at *p* < 0.05 for all analyses.

## Results

### Participant Characteristics

As shown in Table 1, no significant differences were observed between two groups for age, height, weight, body mass index (BMI), and resting HR (| *ts*| < 1, *ps* > 0.29). Participants in the HF group had significantly higher VO_2_max than those in the LF group [*t*(60) = 11.52, *p* < 0.001]. Moreover, 12 HF participants who underwent the fMRI also had significantly higher VO_2_max than those in 12 LF participants [49.16 ± 1.88 ml/kg/min > 42.52 ± 0.99 ml/kg/min, *t*(22) = 10.79, *p* < 0.001]. Such results indicate that the HF group had a better CRF level as compared to the LF group, which further confirms that the grouping based on CRF level was appropriate in this study.

**TABLE 1:** Descriptive data showing a comparison of the participants’ demographic and physical characteristics in the two fitness groups (M ± SD).

Variable	High-fit group	Low-fit group	*t*-test
Age (years)	20.32 ± 0.75	20.35 ± 0.61	*n.s.*
Height (cm)	162.31 ± 4.86	161.50 ± 4.79	*n.s.*
Weight (kg)	54.60 ± 5.24	55.80 ± 7.55	*n.s.*
BMI (kg/m2)	20.70 ± 1.50	21.29 ± 2.63	*n.s.*
Heart Rate rest	81.81 ± 12.51	84.94 ± 14.45	*n.s.*
VO2max (ml/kg/min)	54.28 ± 6.32	38.73 ± 4.06	***

*BMI, body mass index; VO_2_max, maximum oxygen consumption; n.s., not significant; ^∗∗∗^*p* < 0.001.*

### Behavioral Performance

For the behavioral performance in the Stroop task, results were presented in Table 2. Given that the high accuracy was observed across all conditions, RT was finally selected for subsequent analyses. We found significant main effects of Condition [*F*(1, 60) = 23.47, *p* < 0.001, η_p_^2^ = 0.28], with the following gradient: Congruent (784.36 ms) < Incongruent (819.43 ms). Significant main effects of Session were observed in both incongruent condition [*F*(1,60) = 5.59, *p* = 0.02, η_p_^2^ = 0.09] and congruent condition [*F*(1,60) = 9.16, *p* = 0.004, η_p_^2^ = 0.13], with emergence of significantly decreased RT after acute aerobic exercise. Results from the Post-hoc analyses further indicate that this positive effect was mainly contributed by the LF group. In the incongruent condition, significantly decreased RT after exercise was only observed in the LF group as compared to post-rest session [*t*(1,30) = −2.37, *p* = 0.02], but not in the HF group [*t*(1,30) = −0.86, *p* = 0.40]. Similarly, in the congruent condition, there was a significant decrease in RT after exercise in the LF group as compared to post-rest session [*t*(1,30) = −2.64, *p* = 0.01], whereas only a decreasing trend on this outcome was observed in the HF group [*t*(1,30) = −1.71, *p* = 0.09]. However, the main Group effect and the Group × Session interaction effect were not significant in both incongruent and congruent conditions (*Fs* < 0.60, *ps* > 0.44).

**TABLE 2:** Behavioral performance in the Stroop color task (M ± SD).

	High-fit group		Low-fit group	
		
	Control	Exercise	Control	Exercise
**Accuracy**				
Incongruent	0.97 ± 0.03	0.97 ± 0.06	0.97 ± 0.04	0.97 ± 0.03
Congruent	0.97 ± 0.03	0.96 ± 0.05	0.97 ± 0.05	0.97 ± 0.05
**Reaction time (ms)**				
Incongruent	848 ± 109	834 ± 113	821 ± 136	776 ± 132
Congruent	808 ± 116	780 ± 120	793 ± 118	756 ± 129

### fMRI Results

**FIGURE 2:**
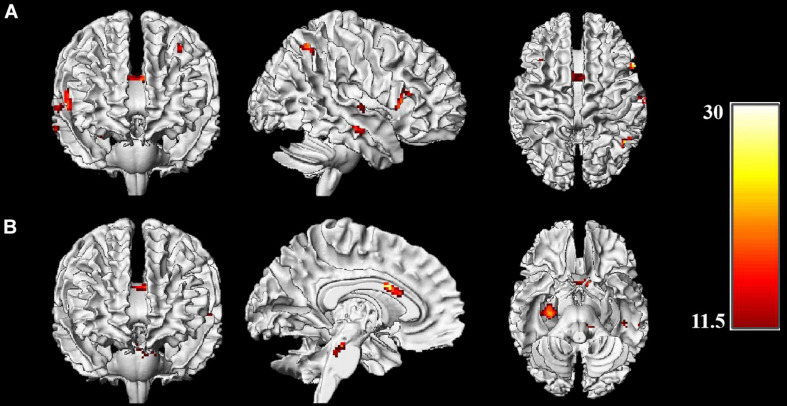
Brain activations of Session (exercise and control) × Group (HF, LF) interactions. **(A)** Whole-brain analysis on incongruent condition; **(B)** Whole-brain analysis on congruent condition. Threshold: *p* < 0.001, uncorrected, *k* > 22. Color bar represents the *F*-value of the interaction analysis.

To determine whether the effects of acute exercise on EF were influenced by CRF level (HF and LF groups), the interactions of Session (post-exercise and post-rest) and Group (high-fit and low-fit) were conducted on brain activation both in incongruent condition and congruent condition. In the incongruent condition, significantly activated brain regions are observed as follow: right dorsolateral prefrontal cortex (rDLPFC), right inferior parietal lobule, right superior and middle temporal gyrus, right hippocampus, left dorsolateral prefrontal cortex (lDLPFC), left superior parietal lobule, and ACC (Figure 2A and Table 3). In the congruent condition, significantly activated brain regions are observed as follow: the right hippocampus and left middle temporal gyrus (Figure 2B and Table 4). These findings indicate the differential effects of acute aerobic exercise on EF between HF and LF groups.

**TABLE 3:** Brain activation of the Session × Group interaction in incongruent condition.

		Peak Activation				
		
	Region	*X*	*Y*	*Z*	*F*-value	Voxels
R	Dorsolateral Prefrontal cortex	56	22	12	18.11	98
R	Inferior Parietal Lobule	46	−56	54	21.26	69
R	Middle Temporal gyrus	66	−14	−10	15.82	53
R	Superior Temporal gyrus	60	−14	4	14.35	25
R	Hippocampus	30	−18	−16	19.21	26
L	Dorsolateral Prefrontal cortex	−36	26	50	16.94	54
L	Superior Parietal Lobule	−38	−62	56	14.40	24
	Anterior Cingulate cortex	−4	8	26	20.31	46

*The peak activations are labeled in MNI coordinates. Threshold: *p* < 0.001, uncorrected, *k* > 22. L, left; R, right.*

**TABLE 4:** Brain activation of the Session × Group interaction in congruent condition.

		Peak Activation				
		
	Region	*X*	*Y*	*Z*	*F*-value	Voxels
R	hippocampus	30	−20	−12	20.67	126
L	Middle Temporal Gyrus	−54	−32	6	16.64	66

*The peak activations are labeled in MNI coordinates. Threshold: *p* < 0.001, uncorrected, k > 22. L, left; R, right.*

ROI analysis was conducted to further confirm the differential effects of exercise on EF (ACC, left DLPFC, and right DLPFC) between two groups with post hoc comparisons. First, beta values were drawn from three key brain regions associated with EF. Second, as all ROIs showed significant Session × Group interaction in the whole-brain analysis, thus we performed the paired sample *t*-test and the independent sample *t*-test on the beta values for the HF and LF groups.

**FIGURE 3:**
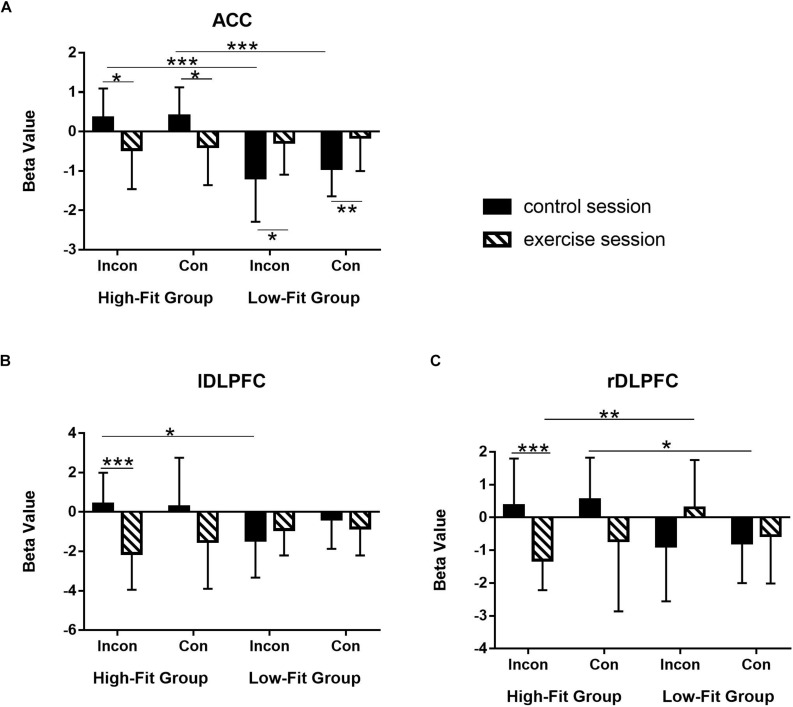
Differentiated effects of aerobic exercise in terms of cardiorespiratory level on the brain activations in three ROIs. **(A)** Anterior cingulate cortex (ACC); **(B)** left dorsolateral prefrontal cortex (DLPFC); **(C)** right DLPFC. Bar represents beta value in parameter estimation. Error bar represents the standard deviation. ***p < 0.001; **p < 0.01;*p < 0.05.

For the ACC (Figure 3A), HF group showed significantly decreased activation after acute-exercise compared to the post-rest control session [incongruent: *t*(11) = −2.86, *p* = 0.02; congruent: *t*(11) = −2.21, *p* = 0.05], whereas LF group shows significantly increased activation [incongruent: *t*(11) = 2.63, *p* = 0.02; congruent: *t*(11) = 2.94, *p* = 0.01]. In addition, HF group showed significantly greater brain activation than LF group [incongruent: *t*(22) = 3.94, *p* = 0.001; congruent: *t*(22) = 4.60 *p* < 0.001] in post-rest session; whereas for the post-exercise session, no significant difference on this brain region was observed between the two groups (*ps* > 0.05).

For the left DLPFC (Figure 3B), HF group showed significantly decreased activation after acute-exercise compared to the post-rest control session in the incongruent condition [*t*(11) = −4.85, *p* = 0.001]; It failed to reach statistical significance in the congruent condition [*t*(11) = −1.99, *p* = 0.07]. No significant difference between two sessions was found in the LF group [incongruent: *t*(11) = 0.98, *p* = 0.35; congruent: *t*(11) = −0.74, *p* = 0.48]. In addition, for the post-rest session, HF group shows significantly greater activation than LF group in the incongruent condition [*t*(22) = 2.54, *p* = 0.02], whereas for the post-exercise session, HF group was slightly lower than that of the LF group in the incongruent condition (*p* = 0.08).

For the right DLPFC (Figure 3C), HF group also showed significantly decreased activation after acute-exercise compared to the post-rest control session in the incongruent condition [*t*(11) = −4.59, *p* = 0.001] but not in the congruent condition [*t*(11) = −1.65, *p* = 0.13]. No significant difference was found in the LF group [incongruent: *t*(11) = 1.69, *p* = 0.12; congruent: *t*(11) = 0.52, *p* = 0.61]. For the post-rest session, HF group shows significantly higher activation than LF group in both the congruent condition [*t*(22) = 2.56, *p* = 0.02] and the incongruent condition (*p* = 0.06), whereas for the post-exercise session, HF group shows significantly lower activation than LF group in the incongruent condition [*t*(22) = −3.21, *p* = 0.004].

### Brain and Behavior Correlation

Pearson correlation analysis was conducted to explore the relationship between ACC and DLPFC. For HF group, there were a significant positive correlation between activation of the rDLPFC and lDLPFC for the post-rest session (*p* = 0.04, *r* = 0.599). Similarly, the positive correlation between activation of the rDLPFC and lDLPFC was still significant (*p* = 0.026, *r* = 0.636) in terms of the post-exercise session. In addition, significant positive correlations were observed between activation of the ACC and lDLPFC in HF group (*p* = 0.018, *r* = 0.665) in terms of the post-exercise session. However, no significant correlation was observed in LF group across all conditions.

Partial correlation analysis was conducted to explore the relationship between behavioral performance and brain activation. In the LF group, significant negative correlations (*p* = 0.05, *r* = −0.68) for the post-exercise session were observed between activation of the ACC and RT in the congruent condition when controlling for their baseline data. However, a marginally significant negative correlations (*p* = 0.09) for the post-exercise session were observed between activation of the lDLPFC and RT in the incongruent condition when controlling for their baseline data. However, no significant correlation was observed in HF group across all conditions.

## Discussion

In the present study, we investigated whether acute exercise-induced changes of behavioral performance in an EF task and corresponding changes in functional brain activation patterns are a function of CRF level. At the behavioral level, our results revealed a significant main Session effect for RT. In particular, we observed that the LF group exhibited faster RT after 30-min exercise session in both the congruent and incongruent condition, while the performance in the HF group did not change significantly in both conditions. These results showed that acute exercise could improve the Stroop performance, which supported a benefit of acute exercise on the EF. However, we did not notice a significant between-group difference in RT. Our findings add to the controversial literature in which mixed findings with respect to behavioral improvements have been reported. In this context, one study reported cognitive improvements regardless of CRF level (Chang et al., 2014a), whereas another study noticed cognitive improvements only in group of low-fit individuals (Li et al., 2019). However, in young adults there is some evidence showing that increased efficiency of brain functioning arising of a higher CRF level can be observed without an improvement of behavioral performance (Themanson and Hillman, 2006; Kamijo et al., 2010). Speculatively, the positive effects of a higher level of CRF on behavioral performance may only emerge when the cognitive task is more difficult (Voss et al., 2011). This, in turn, buttresses the importance of utilizing neuroimaging techniques such as fMRI to elucidate acute exercise-induced changes on neurophysiological level that are not readily observable at the behavioral level.

In the current study, we observed at the neurophysiological level different activation patterns between the HL group and the LF group in brain areas associated with EFs such as the ACC and DLPFC. In particular, the activation of ACC in the HF group was significantly decreased after aerobic exercise in the congruent and incongruent condition, while the opposite brain activation pattern was observed in the LF group. Given that the ACC involved in the monitoring of response conflict (Banich et al., 2000; Hazeltine et al., 2000; Kerns et al., 2004) and plays an important role in attentional control (Liddle et al., 2001) as well as response selection (Milham et al., 2001), the decreased ACC activity in the HF group after exercise might indicate that the demand of conflict control was reduced, leading to a lower recruitment of the ACC. This finding implied that moderate-intensity aerobic exercise might improve the ability to effectively allocate cognitive resources in the HF group. This assumption is, at least, partly underpinned by the fact that a decrease in brain activation in the ACC in our study did not alter behavioral performance in the Stroop task. Moreover, these results are consistent with findings of previous studies which noticed a link between higher levels of CRF and reduced ACC activity (Colcombe et al., 2004; Krafft et al., 2014).

The higher activation of the ACC in the LF group suggests that individuals with a relatively low CRF level are able to recruit more cognitive resources in response to the acute bout of aerobic exercise. Given that we observed improvements in behavioral performance (e.g., decreased RTs) in LF individuals, the increased ACC activity may be attributed to one neurophysiological process that boosts cognitive performance in these cohort. Such explanation is supported by the partial correlation results indicating significant negative correlations between activation of the ACC and reaction time.

In this study, the Stroop task was also found to activate the bilateral DLPFC when we examined the Group by Session interaction effects. It first confirmed that the DLPFC plays a crucial role in the execution of EF-related tasks, which is consistent with previous neuroimaging studies using the same Stroop task (MacLeod and MacDonald, 2000; MacDonald et al., 2000; Aron et al., 2014; Byun et al., 2014; Yanagisawa et al., 2010). Specifically, the DLPFC was found to be associated with cognitive control, attention control and response inhibition (Milham et al., 2002; Prakash et al., 2009; Voelcker-Rehage et al., 2011; Chaddock et al., 2012). Importantly, the activation of DLPFC in the HF group was decreased after the acute aerobic exercise in comparison with the control session; whereas no changes were observed in the LF group. However, the decreased DLPFC activities did not lead to a reduced cognitive control indexed by the unchanged behavioral performance. Hence, it is likely that the acute exercise enhances the efficiency of resource allocation of response inhibition processes in the HF individuals which is probably reflected in decreased DLPFC activity. Such explanation is further supported by the positive correlation result between ACC and lDLPFC after exercise in the HF group indicating the functional connectivity between ACC and lDLPFC in the HF group increased after exercise making resource allocation more efficiently. In contrast, in LF individuals a more pronounced activation in right and left DLPFC was noticed in the incongruent task condition. It suggests that an acute aerobic exercise enables LF individuals to allocate more cognitive resources to the DLPFC in support of promoting inhibition processes which are perhaps mirrored, on behavioral level, in faster RT. Such explanation is supported by the partial correlation result; that is, marginally significant negative correlations between activation of the left DLPFC and reaction time. This assumption is in line with findings of previous studies reporting that in younger adults an acute bout of aerobic exercise leads to a higher task-related DLPFC activation which is linked to improved cognitive performance (Byun et al., 2014; Kujach et al., 2018).

The current study extends our understanding of how the EF can be benefited from acute exercise in different aspects. First, present work highlights the exercise effect on the inhibit control ability measured by the Stroop color task. EF is a set of cognitive processes including working memory, inhibit control, and shifting (Etnier and Chang, 2009; Funahashi and Andreau, 2013). Our previous work has tested the exercise effect on the working memory ability by adopting the N-back paradigm and highlighted the contribution of the ACC and the right cerebellum (Li et al., 2019). Second, our finding reveals dissociated effects between high and low fitness individuals. Specifically, low fitness individuals improved their behavioral performance with equivalent brain activities, whereas high fitness individuals maintained their behavioral performance with reduced brain activities, so as in an energy-saving way. Third, we further provide a neural mechanism to explain how acute exercise promotes EF in high fitness individuals in such an energy-saving way. It is likely that the increased functional connectivity between ACC and lDLPFC in the HF group may contribute to making resource allocation more efficiently.

There are several limitations that should be noted. First, cardiorespiratory fitness (CRF) level determined by the YMCA protocol in this study was recognized as an estimation method from heart rate, which might produce less accurate results than maximal exercise tests (e.g., the exhaled gas analysis). We chose to adopt this method based on a cross-validation study examining the YMCA test among adults, which demonstrated a moderately high significant correlation between the YMCA predicted VO_2_max and the criterion measure (Beekley et al., 2004). Although it was proved to be a useful method for grouping, we admit that other assessments of CRF levels, including using the exhaled gas analysis or other indicators (such as lactate and ventilatory thresholds), might also be considered to produce more accurate results for grouping. Second, the EF was usually measured by the contrast of the incongruent and congruent conditions. We did perform this contrast analysis on our fMRI data. However, it resulted in a very weak brain activation over the inferior parietal lobule which did not survive under the threshold corresponding to the interaction of group and time. Thus, we conducted fMRI analysis separately on the incongruent and congruent conditions. These results reflected the general speed of information processing during the Stroop task and assessed the reallocation of the cognitive source among brain networks. Lastly, the neuroimaging results were based on merely 24 participants in this study; that is a relatively small sample size. Clearly, it will be important in future studies to increase the sample size to reveal comprehensively the neural mechanism underlying the fitness-dependent effect of acute exercise on EF.

## Conclusion

In the current study, we investigated whether acute exercise-induced changes in EF performance and changes in corresponding neurophysiological processes (i.e., functional brain activation changes) are influenced by the individual CRF level. In summary, from a cognitive neuroscience perspective, our findings support the influencing role of CRF level on acute exercise-induced changes in EF and provides preliminary evidence for neural correlates associated with fitness-dependent effects.

## Data Availability Statement

The raw data supporting the conclusions of this article will be made available by the authors, without undue reservation.

## Ethics Statement

The studies involving human participants were reviewed and approved by the Institutional Review Board of the East China Normal University and all study procedures met the guideline of the Declaration of Helsinki. The patients/participants provided their written informed consent to participate in this study.

## Author Contributions

LL: conceptualization and data curation. JC, LL, and CW: formal analysis. LL and JC: investigation and project administration. JC, LL, CW, LZ, FH, NM, CJ, XC, QY, SP, and YZ: methodology. JC, LL, CW, and LZ: writing – original draft. JC, CW, LZ, FH, SP and LL: writing – review and editing. All authors: read and agreed to the published version of the manuscript.

## Conflict of Interest

The authors declare that the research was conducted in the absence of any commercial or financial relationships that could be construed as a potential conflict of interest.

## Footnotes

1 http://www.fil.ion.ucl.ac.uk/spm/software/spm8/

2 http://marsbar.sourceforge.net
